# Magnitude, associated risk factor and awareness of predisposing factors, treatment, and prevention of overweight/obesity among adults in North Wollo zone, Northeast Ethiopia

**DOI:** 10.3389/fnut.2025.1502680

**Published:** 2025-06-13

**Authors:** Aychew Kassa Belete, Yeshiwas Jemberu Asefa, Birhan Ambachew Taye

**Affiliations:** ^1^Department of Sport Science, Faculty of Natural and Computational Science, Woldia University, Woldia, Ethiopia; ^2^Department of Statistics, Faculty of Natural and Computational Science, Woldia University, Woldia, Ethiopia

**Keywords:** overweight, obesity, awareness, prevention, risk factors

## Abstract

**Aim:**

Bad eating habits and an insufficient amount of body activity can contribute to obesity and overweight. This study looked at the prevalence, related risk factors, awareness of predisposing factors and also the avoidance and management of overweight and obesity in North Wollo, Northeast Ethiopia.

**Methods:**

From January 2022 to January 2023, A cross-sectional community survey was carried out with 1084 respondents aged 18 and above in North Wollo, Northeast Ethiopia. A multi-stage sampling technique was employed to recruit participants for the quantitative study, while a convenience sampling technique was employed for the qualitative study. A structured questionnaire that had been pre-tested was utilized to gather quantitative data, while qualitative data was collected using an interview. Body mass index was used to determine overweight and obesity. We employed ordinal logistic regression to examine the relationship between predictors and dependent variables. A *p*-value of 0.05 was statistically significant at 95% confidence.

**Results:**

Overweight/obesity affected 21.3% of the population. Some risk factors were known to the majority of participants, such as excessive saturated fat consumption (52.4%) and a lack of regular physical activity (82.9%). However, a large proportion of respondents were not aware about family history (84.0%), age (57.9%), low intake of vegetables (63.0%), low intake of fruit (77.0%), low socioeconomic status (72.0%), smoking (82.5%), medication (58.0%), stress (82.9%), and lack of sufficient sleep (57.9%) were risk factors for overweight and obesity. The mass of respondents was aware of several methods for preventing overweight and obesity, such as limiting saturated fat consumption (52.4%) and engaging in regular physical activity (82.9%). However, a large percentage of respondents were unaware about getting enough sleep (57.9%), reducing stress (82.9%), managing medications (58.0%), quitting smoking (82.5%), having a good socioeconomic status (72.0%), high intake of fruit (77.0%), and high intake of vegetables (63.0%) could help prevent overweight and obesity. The majority of responders were aware that regular exercise helps with treatment strategies for fatness. However, the vast majority of responders were unaware that well-informed nutrition, weight loss medicine, and weight loss surgery were therapeutic options for overweight and obesity. High levels of stress (*p* = 0.000), female gender (*p* = 0.000), increasing age (0.000), physical inactivity (*p* = 0.000), low intake of fruit and vegetables (*p* = 0.000 and *p* = 0.012), smoking (*p* = 0.000), excessive use of saturated fat (0.043), and alcohol consumption (*p* = 0.000) all significantly increased the risk of over fatness.

**Conclusion:**

According to the findings of this study, one-fifth of adults are overweight or obese. The majority of participants were unaware of typical risk factors for overweight/obesity, as well as prevention and treatment approaches for unhealthy habits and lack of physical activity.

## Introduction

Overweight is having a BMI of between 25 and 29.9 kg/m^2^, whereas obesity is defined as having a BMI of 30 kg/m^2^ or higher (1). Both disorders are characterized by a fat buildup that is abnormal or excessive in the body. BMI is a straightforward measurement and a widely used to estimate overall body composition within a population (2).

Over the last few years, prevalence of obesity has risen globally (3). According to a report from the World Health Organization (WHO), more than 1.9 billion persons were overweight in 2016, with over 650 million being obese (4). This means that globally, around 13% of adults were obese in 2016 (5). Obesity rates vary greatly across areas and countries. Obesity is more common in high-income countries such as the United States, Canada, and Australia than in countries with low and moderate incomes (6). However, the incidence of obesity is fast increasing in many countries with low and moderate incomes, particularly in metropolitan areas (7).

Overweight and obesity have also become more prevalent in Africa in recent years. According to the WHO, over 38% of persons in Africa were overweight or obese in 2016 (8). In North Africa, the occurrence of obesity is generally higher than in other regions, with countries such as Egypt and Tunisia having obesity rates of over 30% (9). Obesity prevalence in Ethiopia was found to be relatively low compared to other Sub-Saharan African countries, according to a 2016 study published in the International Journal of Environmental Research and Public Health. In accordance with the survey, the incidence of obesity among women was 5.9 and 1.7% among males (10). However, it is crucial to highlight that the frequency of obesity may have increased in recent years as a result of changes in lifestyle and nutrition patterns, urbanization, and other factors. Furthermore, there may be differences in obesity rates between different locations and populations within Ethiopia. Age, gender, family history, an unhealthy lifestyle, and physical inactivity have either direct or indirect effects on obesity (11–13). Recent research by Kumer A. suggests that regular physical activity, eating more fruits and vegetables, lowering saturated fat, quitting smoking, and managing stress all play important roles in the prevention of overweight/obesity (14, 15).

Sedentary life style have been linked to a variety of health issues, including cardiovascular disease, musculoskeletal illnesses, cancer, lung disorders, mental disorder, spinal deformity, and metabolic disorders (16, 17). Several factors, such as awareness, attitudes, sociodemographic traits, behavioral patterns, and biomedical factors, can play a role in the development of overweight and obesity Biomedical factors that can be measured in relation to overweight and obesity include body mass index (BMI), which estimates body composition based on height and weight, and waist circumference, a measure of abdominal fat that indicates risk for metabolic conditions (18). Despite increasing these risk factors, limited data can be found about the prevalence and awareness of the population regarding risk factors and the prevention and treatment of obesity/overweight in Ethiopia, Although there is an increase in sedentary life and unhealthy diet, alcohol intake, and smoking, which can cause different metabolic disorder in Ethiopia (19, 20), there is no single study that has assessed the community’s awareness of risk factors and the prevention and treatment of obesity/overweight. As a result, this study was aimed at assessing the prevalence and awareness of risk factors and the prevention and treatment of obesity among adults who live in Northeast Ethiopia.

## Materials and methods

### Study setting, design, period, and population

From January 2022 to January 2023, a community-based cross-sectional survey was conducted among 1,084 persons aged 18 and above in North Wollo, Northeast Ethiopia. Both qualitative and quantitative research approach was applied. North Wollo is one of Amhara National Regional State’s eleven Zones. North Wollo was named after the past region of Wollo. South Wollo borders North Wollo on the south, South Gondar on the west, Wag Hemra on the north, Tigray Region on the northeast, and Afar Region on the east. Woldia is a province in the North Wollo Zone. Woldia is the capital of the north Wollo Zone. According to the Central Statistics Agency of Ethiopia’s (CSA) 2007 Census, Total population of North Wollo is 1,500,303 people, with 752,895 men and 747,408 women. The greater part of the population adhered to Ethiopian Orthodox Christianity, with 80.49% identifying as such, while 18.46% identified as Muslim. The populations were all adults aged 18 and over living in the North Wollo Zone.

### Inclusion and exclusion criteria

This study included all persons aged 18 and above who had lived in the district for 6 months or more; those with significant sickness, mental illness, refusal to reply to the questionnaire, and women with known pregnancy were omitted.

### Sample size determination and sampling technique

In this research, multi- stage, stratified probability sampling technique was applied. Of the 1,838,753 residents in the North Wollo zone as estimated in 2015, 726,657 were adults age above 18 years. From target population of 726,657, we used 1,084 respondents using the Cochran formula. The probability of success and failure will take 0.5 each and margin of error was taken 3%.


$$n_{0}=\frac{z_{\frac{\alpha }{2}}\,p\,q}{d^{2}}$$

$$n=\frac{n_{0}}{1+\frac{n_{0}}{N}}$$

Using this formula total sample size is determined below:


$$n_{0}=\frac{1.96^{2}*0.5*0.5}{0.03^{2}}$$

$$=\frac{0.9604}{0.0009}$$

=1084

The final calculated sample size was 1,084. A two-stage sampling design was used to select study participants. First stage, The sample size was allocated to each of the selected kebeles using probability proportional to size allocation. Second stage, 1,084 households meeting the inclusion criteria were selected using systematic random sampling. The first household was chosen using a lottery method, and subsequently, every tenth household was recruited. If there was more than one eligible adult in a household, one individual was selected at random using the lottery method. Ultimately, a total of 1,084 adults participated in the study.

### Definitions and measurement

According to the Communicable Disease Control categorical classification, the respondents

were classified based on their BMI as follows (21).

*Overweight*: BMI = 25–29.9 kg/m^2^

*Obese*: BMI ≥ 30 kg/m^2^

#### Awareness

Participants’ self-reported beliefs and knowledge regarding obesity risk factors, prevention, and treatment plans. To assess this, a structured questionnaire was utilized, featuring statements to which participants responded with “yes” or “no.” Responses were categorized into two groups: “aware” for those who answered “yes,” and “not aware” for those who answered “no” (22).

#### Physical activity levels

Participants physical activity level can be categorized as follows: Sedentary individuals engage in minimal movement, typically spending over 6 h per day in activities like sitting or lying down, and do not meet the recommended guidelines of at least 150 min of moderate-intensity or 75 min of vigorous-intensity exercise per week. Fairly active individuals participate in moderate physical activity for at least 150 min or vigorous activity for at least 75 min weekly, often incorporating a mix of both but not consistently reaching higher levels of intensity. Active individuals exceed the recommended guidelines, engaging in more than 300 min of moderate-intensity or over 150 min of vigorous-intensity exercise weekly, often participating in structured workouts or various physical activities regularly (23).

#### Adequate sleep

In this study, adequate sleep defined based on established guidelines from the National Sleep Foundation, adults aged 18–64 years should sleep 7–9 h per night for optimal health Respondents who report sleeping between 7 and 9 h per night was answer “yes,” while those who sleep less than 7 h per night was respond with “no” (24).

### Data collection instruments and procedures

A structured questionnaire adapted from the World Health Organization instrument for step wise surveillance (WHO STEPS) of chronic disease risk factors (25). The data was gathered using a structured questionnaire. The same questionnaire was prepared in English and translated to Amharic and then back to English to maintain its consistency. It is contained of four parts. The first section focuses on the socio-demographic characteristics of the participants, including age, sex, education level, marital status, monthly income, and employment status. The second section evaluates the respondents’ behavioral, dietary, and biomedical characteristics, such as smoking habits, alcohol consumption, physical activity, and vegetable intake. The third section of the instrument included 11 statements designed to evaluate participants’ perceptions and knowledge about overweight/obesity and its associated risk factors, demonstrating a strong internal consistency with a Cronbach’s alpha of 0.79. The fourth section comprised thirteen statements aimed at assessing participants’ understanding of the prevention and treatment of overweight/obesity. The internal validity was also examined by SPSS and resulted well with a Cronbach’s alpha value of 0.85.

Data was gathered by a team of 22 trained data collectors. They received training on the study’s objectives, goals, and ethical considerations related to data collection. During the interviews, they conducted anthropometric measurements, including weight, height, and body mass index (BMI). A pretest was administered to 5% of the participants prior to data collection. The cross-validity of the questionnaire was evaluated by two experts in the field. Following minor adjustments based on their feedback, which enhanced the clarity and ease of the questionnaire, actual data collection was conducted daily in working days. The supervisors oversaw the entire data collection process, closely monitoring the quality, clarity, and integrity of the procedures. For the qualitative component, a structured open-ended interview guide was utilized. Conducting the interviews in the participants’ native language (Amharic) facilitated simpler and more effective responses. Trained and experienced professionals collected qualitative data through audio recordings and note-taking. The BMI of respondents was calculated by dividing their weight in kg by their height in m^2^.

A typical beam balance, which is used to assess weight in medical settings, was employed for the weight measurement. Prior to each participant’s measurement, the scale’s pointer was zeroed. Each person shed a hefty clothing. He or she was standing upright in the middle of the balancing platform without any support. The weight was measured to the closest 0.1 kg.

According to normal anthropometric protocols, height was measured using a wooden height-measuring board with a sliding head bar. After being instructed to take off their shoes and stand erect, the participants were put in the Frankfurt plane with their knees straight and their feet together. The occipital region of the back of the head, the heels, buttocks, and the shoulder blades touched the stadiometer’s vertical stand, and readings were taken to the closest 0.1 cm.

### Data entry and statistical analysis

The data was entered, coded, and analyzed using the Statistical Package for Social Sciences version 26 after it was checked for quality and clarity. A Q-Q plot and histogram were used to assess the data’s normal distribution. For categorical variables, descriptive findings were reported using frequency and percentage. Following the usage of the multi-co-linearity enters approach; the goodness of model fit test and the parallel line assumption test were performed. The connection between overweight/obesity and other independent risk variables was determined using ordinal logistic regression analysis. A *p*-value of 0.05 with a 95% confidence interval was considered statistically significant.

### Research ethics approval and consent form

The project was ethically authorized by Woldia University’s ethical review board. The Woldia University Institutional Review Board (WDU/IRB) office provided a formal authorization letter with the protocol number WDU/IRB001. Following a briefing on the study’s aims, participants were informed and gave both written and verbal consent forms. Participants in the study were able to offer informed consent and had a thorough knowledge of the study’s objectives. All processes were carried out by relevant standards and regulations based on Helsinki legislation.

## Results

### Socio-demographic character of the participants

This survey included 1,084 participants aged 18 and above, with a response rate of 100%. In terms of age, the majority (53.1%) of respondents are between the ages of 18 and 29, 37.5% are between the ages of 30 and 49, and 9.4% are over 50. Similarly, 65.2% were men and 34.8% were women. As for educational attainment, 14.4% are illiterate, 19.7% have a primary education, 29.2% have a secondary education, and the remaining 36.7% have a college education or above. 33.6% were married, 55.5% were single, 7.7% divorced, and 3.2% were widowed. In terms of occupation, 19.4% were merchants, 42.7% were government employees, 26.9% were students, 8.7% were farmers, and the other 2.3% were involved in other activities. According to respondents’ monthly household income, 18.6% have less than 2,000 ETB, 41.1% have 2,000–4,000 ETB, 16.2% have income 4,000–6,000 ETB, and 24.1% have income greater than 6,000 ETB (Table 1).

**TABLE 1:** Socio-demographic character among adults in Woldia City, Northeast Ethiopia.

Variable	Categories	Frequency	Percentage
Age	18–29	575	53.1%
	30–49	407	37.5%
	50 and above	102	9.4%
Sex	Women	377	34.8%
	Men	707	65.2%
Marital status	Married	364	33.6%
	Single	602	55.5%
	Divorced	83	7.7%
	Widowed	35	3.2%
Educational status	Unable to read and write	156	14.4%
	Primary	214	19.7%
	Secondary	316	29.2%
	Collage and above	398	36.7%
Occupational status	Merchant	210	19.4%
	Government worker	463	42.7%
	Student	292	26.9%
	Farmer	94	8.7%
	Others	25	2.3%
Monthly family income	Less than 2,000 ETB	202	18.6%
	2,000–4000 ETB	445	41.1%
	4,000–6,000 ETB	176	16.2%
More than 6,000 ETB	261	24.1%

### Behavioral, dietary, and biomedical character of respondents’

When it comes to stress levels, the majority of respondents (66.2%) are stress-free. In terms of smoking habits, the vast majority (79.1%) of respondents never smoke. As for alcohol use, 52.5% were current consumers of alcohol. According to the data, 40.6% of people ate fruit at least three times per week. 45.4% of people consume vegetables less than three times each week. Unsaturated oil is utilized by 52.8% of people, regardless of the type of oil they use.

38.9% were very active, regardless of their degree of physical exercise. According to the level of adequate sleep, 51.1% of people got enough sleep. As for family history of obesity, 60.9% of respondents had no family history of obesity (Table 2).

**TABLE 2:** Behavioral, dietary, and biomedical related characteristics among the participants.

Variables	Categories	Frequency	Percentage
Stress	Never	718	66.2%
	Currently	246	22.7%
	Previously	120	11.1%
Type of oil most often used	Unsaturated	572	52.8%
	Saturated	512	47.2%
Adequate sleep	No	530	48.9%
	Yes	554	51.1%
Physical activity level	Sedentary	369	34.0%
	Fairly active	293	27.0%
	Active	422	38.9%
Smoking	Never	857	79.1%
	Currently	75	6.9%
	Previously	152	14.0%
Frequency of drinking alcohol per weak	Never	515	47.5%
	Less than 3	246	22.7%
	More than 3	323	29.8%
Vegetable consumption per a weak	Never	437	40.3%
	Less than 3	492	45.4%
	More than 3	155	14.3%
Fruit consumption per a weak	Never	308	28.4%
	Less than 3	336	31.0%
	More than 3	440	40.6%
Family history of obesity	Yes	424	39.1%
	No	660	60.9%

### Prevalence of overweight/obesity

**FIGURE 1:**
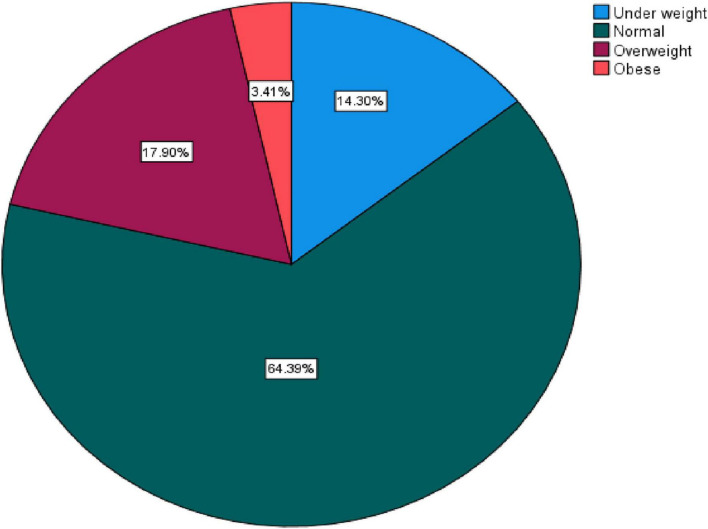
Prevalence of overweight/obesity.

Overweight was prevalent in 17.9% of the population, and obesity was prevalent in 3.4%. Overweight and obesity were present in 21.3% of the population. Adults aged 30–49 years had the highest prevalence of overweight or obesity, followed by adults aged 50 and up. The prevalence of overweight in males was 7.9 and 10.0% in females, obesity in males was 1.65 and 1.75% in females, and the combined prevalence of overweight and obesity in males was 9.55 and 11.75% in females (Figure 1).

### Participant’s knowledge of risk factors for overweight/obesity

The mass of respondents was aware of some risk factors, such as high intake of saturated fat (52.4%) and a lack of adequate physical activity (82.9%). However, the greater part of respondents (84.0%) were unaware that family history (84.0%), age (57.9), low vegetable intake (63.0%), low consumption of fruit (77.0%), low socioeconomic status (72.0%), smoking (82.5%), medication (58.0%), stress (82.9%), and lack of adequate sleep (57.9%) were risk factors for overweight and obesity (Table 3).

**TABLE 3:** The awareness of respondents on unhealthy lifestyle, physical inactivity, and other cardiovascular and overweight/obesity risks.

Variable	Categories	Frequency	Percentage
lack of adequate sleep	Yes	456	42.1%
	No	628	57.9%
Stress	Yes	185	17.1%
	No	899	82.9%
Medication	Yes	455	42.0%
	No	629	58.0%
Lack of physical activity	Yes	899	82.9%
	No	185	17.1%
Smoking	Yes	190	17.5%
	No	894	82.5%
Low socioeconomic status	Yes	300	28.0%
	No	784	72.0%
Low Intake of Fruit	Yes	250	23.0%
	No	834	77.0%
Low Intake of vegetable	Yes	400	37.0%
	No	684	63.0%
High consumption of saturated fat	Yes	568	52.4%
	No	516	47.6%
Age	Yes	456	42.1%
	No	628	57.9%
Family history	Yes	170	16.0%
	No	914	84.0%

### Participant’s knowledge of prevention techniques for overweight/obesity

The mass of respondents was aware of various preventative methods for overweight and obesity, such as limiting saturated fat consumption (52.4%) and engaging in regular physical activity (82.9%). However, the majority of respondents were unaware that adequate sleep (57.9%), stress reduction (82.9%), medication management (58.0%), smoking cessation (82.5%), good socioeconomic status (72.0%), a high intake of fruit (77.0%), and a high intake of vegetables (63.0%) were prevention techniques for overweight and obesity risk (Table 4).

**TABLE 4:** The awareness of respondents on prevention technique overweight/obesity.

Variable	Categories	Frequency	Percentage
Making adequate sleep	Yes	456	42.1%
	No	628	57.9%
Reducing stress	Yes	185	17.1%
	No	899	82.9%
Managing Medication	Yes	455	42.0%
	No	629	58.0%
Being physical active	Yes	899	82.9%
	No	185	17.1%
Stop smoking	Yes	190	17.5%
	No	894	82.5%
Good socioeconomic status	Yes	300	28.0%
	No	784	72.0%
High Intake of fruit	Yes	250	23.0%
	No	834	77.0%
High Intake of vegetable	Yes	400	37.0%
	No	684	63.0%
Low consumption of saturated fat	Yes	568	52.4%
	No	516	47.6%

### Participant’s knowledge of treatment techniques of overweight/obesity

The bulk of respondents (55.4%) were aware of some treatments for overweight and obesity, such as frequent exercise. The majority of respondents, however, were unaware that well-informed nutrition (64.4%), weight reduction medicine (73.9%), and weight loss surgery (68.9%) were treatment options for overweight and obesity risk (Table 5).

**TABLE 5:** The awareness of respondents on treatment technique overweight/obesity.

Variable	Categories	Frequency	Percentage
Regular exercise	Yes	600	55.4%
	No	484	44.6%
Making well informed nutrition	Yes	386	35.6%
	No	698	64.4%
Weight loss medication	Yes	283	26.1%
	No	801	73.9%
Weight loss surgery	Yes	337	31.1%
	No	747	68.9%

### Knowledge of risk factors of overweight/obesity via interview

During the interview stage, a lack of information on risk factors for overweight/obesity was discovered. A high number of respondents stated that they are unaware of the risk factors for obesity, that they do not know about smoking, drinking alcohol, and how saturated fat is related to obesity. However, a 29-year-old woman claimed: “…*The most common risk factor for overweight/obesity is being wealth*…………….” Another 45-year-old guy indicated that he lacked awareness about the risk factors for obesity: “…*In general, I feel that people are predisposed to being overweight or obese as a result of their high level of comfort.*…..” Some respondents who were more aware of the risk factors for overweight and obesity were able to name saturated fat consumption and physical inactivity as risk factors. For example, 23-year-old men claimed: “… *PHYSICAL inactivity exposes for obesity.” Furthermore, increased saturated fat consumption increases the risk of being overweight or obese……..”*

### Association between prevalence of overweight/obesity and other predictors

The association between being obese and other independent variables was demonstrated using ordinal binary logistic regression analysis. Based on the results, age, sex, educational status, marital status, occupational status, monthly family income, smoking, frequency of drinking of alcohol per week, fruit consumption per week, vegetable consumption per a weak, type of oil most often used, physical activity level, level of stress and family history of obesity were significantly associated with obesity. Participants aged 18–29 are 0.071 times less likely to be overweight or obese than those aged 50 and above (AOR = 0.07, 95% CI: 0.04–0.13; *p* = 0.000). Similarly, being male are 0.22 times less likely to obese than female (AOR = 0.22, 95% CI: 0.09–0.50; *p* = 0.000), monthly family income less than 2,000 ETB (AOR = 0.09, 95% CI: 0.04–0.21; *p* = 0.000), low level of stress (AOR = 0.22, 95% CI: 0.12–0.40; *p* = 0.000), Participants who used unsaturated oil the most frequently (AOR = 0.61, 95% CI: 0.38–0.98; *p* = 0.043), those with active physical activity level (AOR = 0.40, 95% CI: 0.25–0.64; *p* = 0.000) those who never smoked cigarettes (AOR = 0.40, 95% CI: 0.25–0.67; *p* = 0.000) those who never drank alcohol (AOR = 0.15, 95% CI: 0.09–0.24; *p* = 0.000) previous family history of obesity (AOR = 4.07, 95% CI: 2.37–6.99; *p* = 0.000) were less likely to have obese. Educational status not more than primary (AOR = 31.22, 95% CI: 13.12–74.29; *p* = 0.000) were more likely to have obese (Table 6).

**TABLE 6:** Association between overweight/obesity and other independent variables.

Variables	Body mass index				COR (95% CI)	AOR (95% CI)	Sig.
	Under weight N (%)	Normal N (%)	Over Wight N (%)	Obese N (%)			
**Age**							
18–29	86(55.5)	407(58.3)	81(41.8)	1(2.7)	0.15(0.10–0.23)	0.07(0.04–0.13)	**0.000**
30–49	58(37.4)	252(36.1)	88(45.4)	9(24.3)	0.22(0.14–0.34)	0.16(0.09–0.29)	**0.000**
>50	11(7.1)	39(5.6)	25(12.9)	27(73.0)		1	
**Sex**						1	
Men	127(81.9)	147(21.1)	85(43.8)	18(48.6)	0.49(0.37–0.63)	0.22(0.09–0.50)	**0.000**
Women	28(18.1)	551(78.9)	109(56.2)	19(51.4)		1	
**Marital status**						1	
Married	135(87.1)	185(26.5)	23(11.9)	21(56.8)	0.04(0.02–0.08)	0.13(0.04–0.38)	**0.000**
Single	16(10.3)	499(71.5)	87(44.8)	0(0.0)	0.20(0.10–0.38)	0.36(0.12–1.08)	0.069
Divorced	1(0.6)	3(0.4)	64(33.0)	15(40.5)	4.84(2.18–10.73)	5.73(2.04–16.05)	**0.001**
Widowed	3(1.9)	11(1.6)	20(10.3)	1(2.7)		1	
**Educational status**						1	
Unable to read and write	20(12.9)	87(12.5)	30(15.5)	19(51.4)	0.90(0.62–1.30)	3.44(1.90–6.22)	**0.000**
Primary	44(28.4)	158(22.6)	10)5.2)	2(5.4)	0.23(0.16–0.34)	31.22(13.12–74.29)	**0.000**
Secondary	46(29.7)	243(34.8)	27(13.9)	0(0.0)	0.32(0.23–0.44)	0.35(0.18–0.67)	**0.002**
College and above	45(20.9)	210(30.1)	127(65.5)	16(43.2)		1	
**Occupational status**						1	
Merchant	2(1.3)	118(16.9)	72(37.1)	18(48.6)	0.95(0.42–2.11)	0.93(0.29–2.98)	0.903
Government worker	16(10.3)	344(49.3)	95(49.0)	8(21.6)	0.37(0.17–0.81)	0.76(0.25–2.26)	0.619
Student	121(78.1)	160(22.9)	9(4.6)	2(5.4)	0.03(0.01–0.07)	0.11(0.03–0.40)	**0.001**
Farmer	12(7.7)	68(9.7)	8(4.1)	6(16.2)	0.18(0.07–0.44)	0.27(0.08–0.88)	**0.030**
Others	4(2.6)	8(1.1)	10(5.2)	3(8.1)		1	
**Monthly family income**						1	
<2,000 ETB	80(51.6)	115(16.5)	5(2.6)	2(5.4)	0.02(0.01–0.04)	0.09(0.04–0.21)	**0.000**
2,000–4,000 ETB	60(38.7)	351(50.3)	28(14.4)	6(16.2)	0.09(0.06–0.12)	0.07(0.03–0.14)	**0.000**
4,000–6,000 ETB	12(7.7)	116(16.6)	39(20.1)	9(24.3)	0.31(0.21–0.46)	0.36(0.19–0.67)	**0.001**
>6,000	3(1.9)	116(16.6)	122(62.9)	20(54.1)		1	
**Stress**						1	
Never	145(93.5)	536(76.8)	29(14.9)	8(21.6)	0.07(0.05–0.11)	0.22(0.12–0.40)	**0.000**
Currently	3(1.9)	103(14.3)	127(65.5)	13(35.1)	1.41(0.92–2.14)	0.88(0.49–1.57)	0.663
Previously	7(4.5)	59(8.5)	38(19.6)	16(43.2)		1	
**Type of oil most often used**						1	
Unsaturated	64(41.3)	465(66.6)	43(22.2)	0(0.0)	0.41(0.32–0.53)	0.61(0.38–0.98)	**0.043**
Saturated	91(58.7)	233(33.4)	151(77.8)	37(100)		1	
**Adequate sleep**						1	
No	98(63.2)	295(42.5)	116(59.8)	21(56.8)	1.05(0.82–1.33)	1.25(0.82–1.89)	0.301
Yes	57(36.8)	403(57.7)	78(40.2)	16(43.2)		1	
**Physical activity level**						1	
Active	106(68.4)	129(18.5)	112(57.7)	22(59.5)	1.11(0.83–1.47)	0.40(0.25–0.64)	**0.000**
Fairly active	43(27.7)	191(27.4)	44(22.7)	15(40.5)	0.99(0.73–1.34)	0.27(0.16–0.46)	**0.000**
Sedentary	6(3.9)	378(54.2)	38(19.6)	0(0.0)		1	
**Smoking**						1	
Never	132(85.2)	594(85.1)	129(66.5)	2(5.4)	0.30(0.21–0.43)	0.40(0.25–0.67)	**0.000**
Currently	7(4.5)	27(3.9)	29(14.9)	12(32.4)	1.82(1.08–3.09)	1.35(0.64–2.84)	0.433
Previously	16(10.3)	77(11.0)	36(18.6)	23(62.2)		1	
**Frequency of drink alcohol per weak**						1	
Never	110(71.0)	365(52.3)	40(20.6)	0(0.0)	0.19(0.14–0.26)	0.15(0.09–0.24)	**0.000**
<3	21(13.5)	152(21.8)	58(29.9)	15(40.5)	0.75(0.54–1.04)	0.18(0.10–0.32)	**0.000**
>3	24(15.5)	181(25.9)	96(49.5)	22(59.5)		1	
**Vegetable consumption per weak**						1	
Never	19(12.3)	283(40.5)	99(51.0)	36(97.3)	1.21(0.83–1.75)	0.89(0.51–1.55)	0.687
<3	118(76.1)	331(47.4)	42(21.6)	1(2.7)	0.24(0.16–0.35)	0.52(0.31–0.87)	**0.012**
>3	18(11.6)	84(12.0)	53(27.3)	0(0.0)		1	
**Fruit consumption per weak**						1	
Never	102(65.8)	75(10.7)	115(59.3)	16(43.2)	1.37(1.01–1.84)	0.39(0.24–0.63)	**0.000**
<3	34(21.9)	234(33.5)	55(28.4)	13(35.1)	1.28(0.96–1.72)	1.42(0.89–2.25)	0.137
>3	19(12.3)	389(55.7)	24(12.4)	8(21.6)		1	
**Family history of obesity**						1	
Yes	29(18.7)	205(29.4)	158(81.4)	32(86.5)	8.06(5.95–10.92)	4.07(2.37–6.99)	**0.000**
No	126(81.3)	493(70.6)	36(18.6)	5(13.5)		1	

COR, crude odds ratio; AOR, adjusted odds ratio. Bold values: indicates a significant variable.

According to Table 6, as for age, those aged 18–29 are 0.071 times less likely to be overweight or obese than those aged 50 and above. In terms of gender, female respondents were more likely than males to be overweight or obese. As for the impact of educational status, those respondents who had a primary level of education were 31.220 times more likely to be overweight or obese than respondents who were college and above educational level, and participants who had a secondary level of education had 0.350 times lower risk to be overweight and obese as compared to those who have collage and above educational level participants.

As for occupational status, participants who were students had 0.112 times lower risk for being overweight and obese as compared to those who have occupational status “others.” In the case of monthly family income, respondents who have a family monthly income of more than 6,000 ETB are more likely to be overweight and obese. As for the impact of stress, respondents who live with stress previously had a greater risk for to be overweight and obese. As for the type of oil most often used, respondents who used unsaturated oil mostly are 0.613 times lower risk of being overweight and obese as compared to saturated oil users.

In terms of adequate sleep, respondents who had good sleep experience (yes) had a lower risk than respondents who did not have a good sleep experience (no). As for physical activity level, participants who have a sedentary level of physical activity were more likely overweight and obese. As for smoking, participants who never smoked had 0.404 times lower risk as compared to previous smokers, and participants who are currently smokers had 1.347 times higher risk for being overweight and obese than previous smokers. As per frequency of drinking alcohol, participants who drank alcohol more than 3 days per week had a higher risk of being overweight and obese. In the case of vegetable consumption per week, participants who never ate vegetables were more likely to be overweight and obese.

As for fruit consumption per week, participants who ate fruit more than 3 days per week had a lower risk of being overweight and obese as compared to participants who never ate fruit. On the impact of family history related to obesity, participants who have a history of obesity in their family were 4.071 times more likely to be overweight and obese than participants who never history of obesity.

## Discussion

The mass of Ethiopian researchers perform studies on the prevalence and risk factors of overweight and obesity (26–28). There is no one published study that has assessed the level of community awareness of risk factors, preventions, and treatments of overweight/obesity in the North Wollo population, based on the authors’ best search approach. As a result, the magnitude and awareness of risk factors, prevention, and treatment of overweight/obesity among adults in north Wollo, Northeast Ethiopia, were investigated in this study. Among the North Wollo zone, the total prevalence of overweight and obesity among adults aged 18 and above was 21.3%. According to our research, the findings achieved are lower than the other studies (as for prevalence) conducted in Ethiopia. As researchers, we suspected that the location where the research was conducted had become a war zone over the previous 3 years. Because, the majority of the residents of North Wolo migrated during Tigray’s invasion. There was a transportation difficulty during the exodus, thus most of the evacuees were forced to walk for many days. Hunger and thirst were also used against them. We are convinced that this scarification is related to the findings of the current investigation. This was discovered to be more than the EDHS report of 2016 (29) and Congo (30), but lower than the result of united state (31), Nigeria (32), Portuguese (33), Turkey (34), and China (35). In addition, it is in line with a study conducted in the Democratic Republic of Congo (36), Benin (36), and Malawi (37).

This study states that a large proportion of the respondents were aware of some risk factors, such as high consumption of saturated fat (52.4%) and lack of physical exercise (82.9%). this result is supported by a previous study (38–40) However, a large percentage of respondents were not aware that family history (84.0%), age (57.9), low intake of vegetables (63.0%), low intake of fruit (77.0%), Low socioeconomic status (72.0%), smoking (82.5%) medication (58.0%), stress (82.9%), and lack of adequate sleep (57.9%) had risk factor for overweight and obesity risk. The mass of the respondents was aware of some prevention techniques of overweight and obesity, such as low consumption of saturated fat (52.4%), and regular physical exercise (82.9%). However, a large proportion of the respondents were not aware that getting adequate sleep (57.9%), reducing stress (82.9%), managing medication (58.0%), stopping smoking (82.5%), good socioeconomic status (72.0%), high intake of fruit (77.0%), and high intake of vegetable (63.0%) had prevention technique for overweight and obesity risk. The majority of the respondents were aware of some treatment techniques of overweight and obesity, such as regular exercise (55.4%). However, the majority of the respondents were not aware that making well-informed nutrition (64.4%), weight loss medication (73.9%), and weight loss surgery (68.9%) had treatment techniques for overweight and obesity risk.

An example of the attitudes of the respondents was made known to the authors of this study through a personal revelation Despite struggles with overweightness, the man known to the authors had become regularly engaged in sport, managing to shed some pounds over time. After 4 months, He visits his family who he had not seen in a long time.

Upon arriving at his family home, his mother embraced him tightly, tears welling in her eyes as she asked, “What happened to you, my son?” Her emotional reaction was rooted in the cultural perception in Ethiopia, where gaining weight is often seen as a sign of comfort and prosperity.

Most of the studies conducted in different countries showed that a prevalence of obesity is observed among women more than men (41, 42). Based on the results of this study, the prevalence of overweight in males was 7.9% and in females was 10.0%, obesity in males was 1.65% and in females was 1.75%. Our research prevails that the prevalence of overweight/obesity is higher in women than men. This finding is supported by studies in Iran (42), Latin America (41), and EDHS reports of 2016 (43). Differently, the studies in united states (31), Turkey (44), Danish (45), and Spanish (46).

The findings in the present study showed a statistical association between age, sex, educational status, monthly family income, smoking, frequency of alcohol conception, vegetable and fruit consumption, level of stress, physical activity level, family history, and overweight or obesity. This is in line with other similar findings in Kerala, India (47), Uganda (48), and Porto (49). On the contrary, people with the lowest income and socioeconomic group were more likely to be obese (50). In addition, participants with the highest income in the study area might be increased food intake of high calories, reduced physical activity, and increased sedentary lifestyles (51).

This study identified the odds of being overweight or obese is increased with increasing age. This finding is in line with other similar findings in Saudi Arabia (1), Great Britain (52), Benin (53), Atlanta (54), and Shanghai (55). This is highly linked to physical inactivity and hormonal change due to becoming older.

Obesity results from energy imbalance: too many calories in, too few calories burned. Several factors influence how many calories (or how much “energy”) people burn each day, among them, age, body size, and genes. But the most variable factor is also the most easily modified the amount of activity people get each day (56). In this study, physical activity was found to be adversely related with obesity and overweight. Physically inactive persons had a greater risk of being overweight or obese than physically active ones. This finding is supported by the study among adults in Swedish (57), Canada (58), South Africa (59), and Ghana (60). This might be explained by the accepted belief that keeping active can help people stay at a healthy weight or lose weight.

Stress has long been associated with changes in dietary preference, food intake, weight gain, and fat accumulation (61). As for the impact of stress, respondents who lived with stress previously had a greater risk for to be overweight and obese. Since, stress was positively associated with obesity in this study. This result is in line with studies in Chicago (62), Australia (63), and Canada (64).

Increased consumption of unsaturated fatty acids at the expense of saturated fatty acids, proteins, and carbs had a positive influence on body weight and obesity (65). As for the type of oil most often used, respondents who used unsaturated oil mostly have 0.613 times lower risk of being overweight and obese as compared to saturated oil users. This result is supported by a study conducted in the United States (66).

The current study’s findings revealed that individuals who used alcohol had a higher risk of becoming overweight or obese than those who did not consume alcohol. This is supported by cross-sectional research done in Welkite, Ethiopia (67), United States (68), and France (69). This might be explained by the understanding that alcohol consumption leads to a positive energy balance because the alcohol they consumed quickly turns to energy; drinking alcohol also seems to trigger impulsive eating behaviors, and alcohol might decrease fat breakdown and can stimulate its synthesis and deposition (70).

The results of this study reveal a concerning gap in knowledge among participants regarding the typical risk factors associated with overweight and obesity, as well as the prevention and treatment strategies for unhealthy habits and physical inactivity, which has significant implications for public health interventions aimed at combating the rising prevalence of obesity and related chronic diseases. The majority of participants’ unawareness of risk factors such as poor dietary habits, sedentary lifestyles, genetic predisposition, and environmental influences suggests that existing public health messaging may not be effectively reaching target populations, potentially leading to a failure to recognize personal risk and a lack of motivation to adopt healthier behaviors. Additionally, the unfamiliarity with prevention and treatment approaches is alarming, as effective strategies, including community-based programs promoting physical activity and nutrition education, are essential for reducing obesity rates; without knowledge of these strategies, individuals may not seek help or engage in preventive measures. Several factors, including socioeconomic status, educational background, and access to health information, may contribute to this observed lack of awareness, along with cultural perceptions surrounding body image and health that influence attitudes towards obesity

### Study strengths and limitations

The utilization of a broader representative sample, as well as the standardized survey technique and measures, can be cited as positives of this study. However, because selection bias may impact the composition of participants, it is difficult to conclude. The study design, a cross-sectional survey approach, limits drawing causal conclusions between the variables studied. As a result, understanding study limitations may be significant in interpreting and applying the findings. More research on these limits in the area will be encouraged.

## Conclusion

The current study can conclude that more than one-fifth of adults are overweight/obese. The majority of the respondents did not have an awareness of overweight/obesity risk factors, prevention, and treatment techniques regarding unhealthy lifestyles and age. Furthermore, living with stress, a family history of obesity, high consumption of saturated fat, alcohol consumption, older age, smoking, inability to consume fruits, and sedentary life were found to have a significant association with the occurrence of overweight/obesity. Future researchers should focus on educational interventions to raise awareness about obesity risk factors and prevention strategies. Longitudinal studies are needed to track changes in awareness and lifestyle behaviors over time. Additionally, exploring the influence of family dynamics, dietary patterns, and cultural contexts can enhance the effectiveness of obesity interventions.

## Data Availability

The datasets presented in this study can be found in online repositories. The names of the repository/repositories and accession number(s) can be found in the article/supplementary material.
